# Immune cell subsets and their gene expression profiles from human PBMC isolated by Vacutainer Cell Preparation Tube (CPT^™^) and standard density gradient

**DOI:** 10.1186/s12865-015-0113-0

**Published:** 2015-08-26

**Authors:** Christopher P. Corkum, Danielle P. Ings, Christopher Burgess, Sylwia Karwowska, Werner Kroll, Tomasz I. Michalak

**Affiliations:** Molecular Virology and Hepatology Research Group, Division of BioMedical Sciences, Faculty of Medicine, Health Sciences Centre, Memorial University, St. John’s, NL A1B3V6 Canada; Novartis Oncology Companion Diagnostics, Cambridge, MA 02139 USA; Present address: Quidel Corporation, San Diego, CA 92130 USA

**Keywords:** Peripheral blood mononuclear cells, BD Vacutainer cell preparation tube, Immune cell subsets, Ficoll density gradient, RNA, DNA, Gene expression analysis, Affymetrix microarray

## Abstract

**Background:**

High quality genetic material is an essential pre-requisite when analyzing gene expression using microarray technology. Peripheral blood mononuclear cells (PBMC) are frequently used for genomic analyses, but several factors can affect the integrity of nucleic acids prior to their extraction, including the methods of PBMC collection and isolation. Due to the lack of the relevant data published, we compared the Ficoll-Paque density gradient centrifugation and BD Vacutainer cell preparation tube (CPT) protocols to determine if either method offered a distinct advantage in preparation of PBMC-derived immune cell subsets for their use in gene expression analysis. We evaluated the yield and purity of immune cell subpopulations isolated from PBMC derived by both methods, the quantity and quality of extracted nucleic acids, and compared gene expression in PBMC and individual immune cell types from Ficoll and CPT isolation protocols using Affymetrix microarrays.

**Results:**

The mean yield and viability of fresh PBMC acquired by the CPT method (1.16 × 10^6^ cells/ml, 93.3 %) were compatible to those obtained with Ficoll (1.34 × 10^6^ cells/ml, 97.2 %). No differences in the mean purity, recovery, and viability of CD19+ (B cells), CD8+ (cytotoxic T cells), CD4+ (helper T cell) and CD14+ (monocytes) positively selected from CPT- or Ficoll-isolated PBMC were found. Similar quantities of high quality RNA and DNA were extracted from PBMC and immune cells obtained by both methods. Finally, the PBMC isolation methods tested did not impact subsequent recovery and purity of individual immune cell subsets and, importantly, their gene expression profiles.

**Conclusions:**

Our findings demonstrate that the CPT and Ficoll PBMC isolation protocols do not differ in their ability to purify high quality immune cell subpopulations. Since there was no difference in the gene expression profiles between immune cells obtained by these two methods, the Ficoll isolation can be substituted by the CPT protocol without conceding phenotypic changes of immune cells and compromising the gene expression studies. Given that the CPT protocol is less elaborate, minimizes cells’ handling and processing time, this method offers a significant operating advantage, especially in large-scale clinical studies aiming at dissecting gene expression in PBMC and PBMC-derived immune cell subpopulations.

**Electronic supplementary material:**

The online version of this article (doi:10.1186/s12865-015-0113-0) contains supplementary material, which is available to authorized users.

## Background

Gene expression microarray analysis is a powerful method that can provide a global picture of the transcriptional activity in a given biological sample at a particular moment in time. Most of the traditional molecular biology methods allow only the investigation of a single or small group of genes, whereas microarrays can determine expression of thousands of genes simultaneously. Many studies have employed microarrays to define gene signatures for diagnostic purposes [1–4] and to predict patients’ response to different treatment regimens [5–9]. Other studies have utilized this technology to recognize molecular mechanisms underlying disease pathogenesis or its progression [10–14]. As with other RNA-based technologies, the results are to a large degree influenced by the quality of starting material and recovered mRNA. Several factors, including sample collection, storage and transportation, manipulation, and extraction of nucleic acids and their preservation methods can affect the integrity of test material [15–18]. Therefore, careful consideration must be given when designing protocols utilizing the above technologies.

Many gene expression profiling studies have used highly valuable biopsy tissue from diseased or healthy individuals. However, such samples may not be readily available and their procurement might be uncomfortable to patients. Peripheral blood mononuclear cells (PBMC) have become a common source of genomic material for microarray studies, mainly due to the simplicity and relative non-invasiveness of acquisition [3, 4, 6, 7, 11–14, 19]. The classical method of PBMC isolation from whole blood applies Ficoll-Paque density gradient centrifugation [20]. Several commercially available products have been developed to simplify PBMC separation. One of the relatively recently developed approaches is the Vacutainer Cell Preparation Tube (CPT) from Becton Dickinson (BD). This method combines a blood collection tube containing sodium citrate anticoagulant and Ficoll-Paque density medium separated by a gel barrier. This results in a single tube system for the collection of whole blood and separation of plasma and PBMC from erythrocytes and granulocytes. Compared PBMC isolation using Ficoll, the CPT method limits both technical variability and sample manipulations, and reduces the processing time. Whereas previous studies have compared the efficacy, quality and functional capacity of PBMC obtained by these two methods [21–23], no evaluations have been done to assess potential differences in the recovery and quality of immune cell subsets and the quality of genetic material derived from these isolated cells.

PBMC are not a homogenous cell population but are constituted by several immune cell types including, among others, B cells (~15 %), T cells (~70 %), monocytes (~5 %), and natural killer (NK) cells (~10 %) [24, 25]. Consequently, the interpretation of microarray results using total PBMC can be challenging because changes in proportion of cell subsets or in the expression of a subset-specific gene can contribute to differences in gene expression observed in total PBMC. Depending on the type of disease or treatment applied, the proportion of individual immune cell subsets in the total PBMC population can vary considerably [26–28] . Similarly, the method of isolation, delays in processing time, and the technique of cryopreservation can all affect subset ratios in total PBMC [18, 23, 29–31]. Another consideration is that significant fluctuations in subset-specific genes, particularly those which characterize a minority cell subset, may be overlooked when the entire PBMC population is examined. This “dilution effect” can be overcome by the isolation of individual immune cell types prior to RNA extraction [32], but techniques that require considerable *ex vivo* handling may significantly impact the quality of subsequent gene expression analysis [16, 33]. Nevertheless, the isolation of specific immune subsets is a highly valuable approach that may identify unique gene expression profiles which are otherwise masked in the whole PBMC population.

In the current study, we have compared the Ficoll-Paque density gradient centrifugation and CPT methods to determine if the more labour-intensive Ficoll technique can be replaced by CPT to isolate PBMC for downstream gene expression studies. We evaluated the recovery and viability of total PBMC purified using both methods, as well as the quantity and quality of extracted RNA and DNA. In addition, we used an in-house procedure to separate pure, multiple immune subsets from PBMC isolated by Ficoll and CPT approaches to assess the recovery and purity of each subset, along with the quantity and quality of extracted nucleic acids. Finally, we compared gene expression of total PBMC and immune subsets from Ficoll gradient and CPT using microarray technology.

## Methods

### Samples and study design

**Fig. 1:**
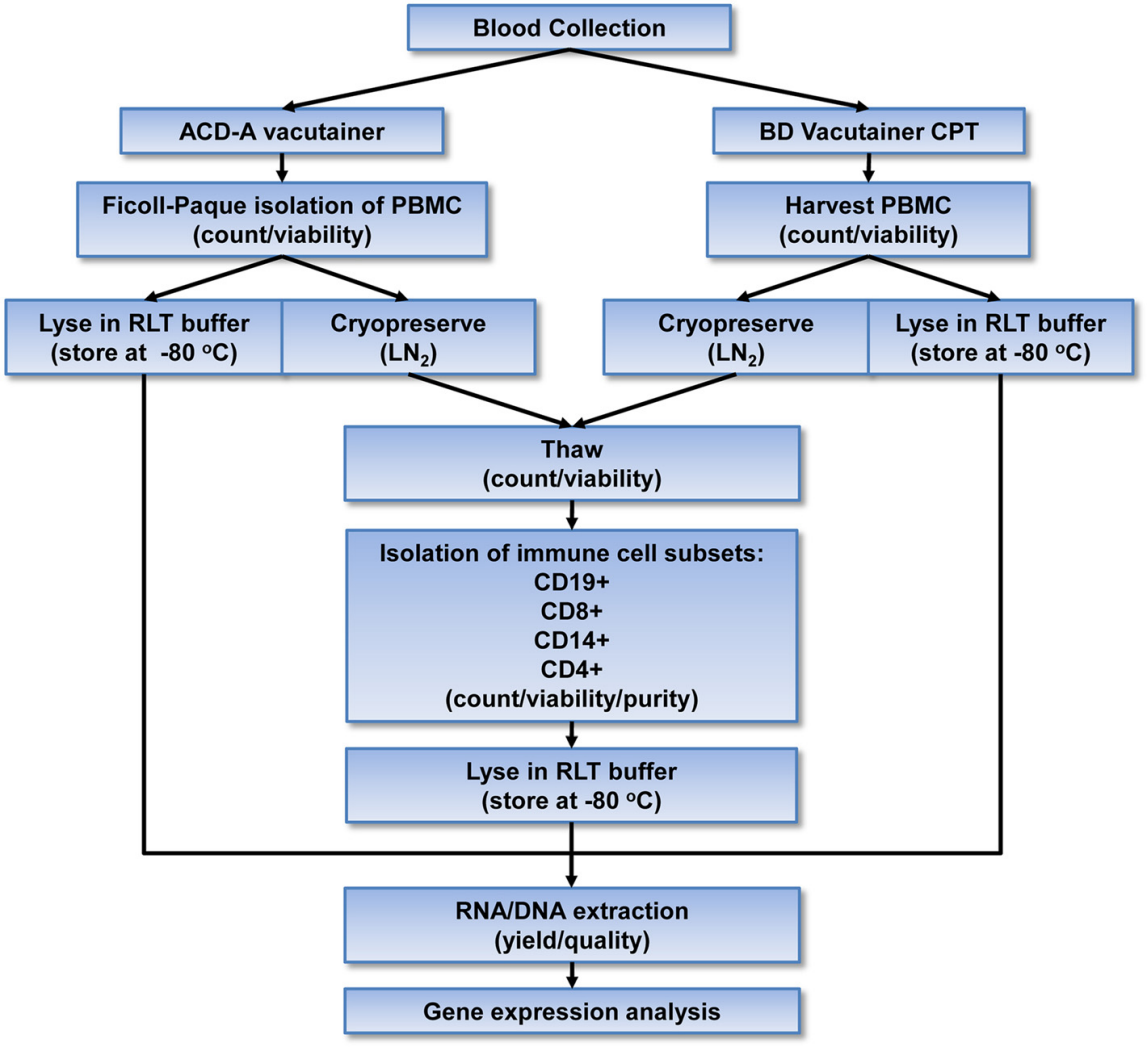
Schematic outline of the study. PBMC from 6 healthy donors were isolated using Ficoll-Paque gradient fractionation or BD Vacutainer CPT protocol, and cell yield and purity were compared. Subsequently, immune cell subsets were separated by positive selection from PBMC obtained by both methods, and yields and viabilities of the subsets were determined and compared. RNA and DNA were extracted from total PBMC isolated by both protocols and from their subsets, and the nucleic acid yield and quality compared. Finally, gene expression analysis of individual immune cell subsets and total PBMC obtained by Ficoll and CPT isolation methods were performed and the results compared. LN_2_, liquid nitrogen

Peripheral blood samples were collected from 6 healthy adult donors. The samples were collected after approval by the institutional Health Research Ethic Authority and signing written informed consent by donors. About 45 ml of blood was obtained from each donor using BD Vacutainer tubes containing acid-citrate-dextrose anticoagulant, solution A (ACD-A; BD) from which PBMC were isolated by Ficoll-Paque gradient centrifugation (see below). PBMC were divided into two approximately equal parts, one for extraction of RNA and DNA, and the other was cryopreserved for isolation of immune cell subsets (see below). In parallel, another ~45 ml of blood from the same donors was collected directly into BD Vacutainer CPT containing 0.1 M sodium citrate and PBMC were isolated following the manufacture’s instruction (see below). The resulting cells were divided into two parts, as above. Subsequently, B cells, CD4+ and CD8+ T lymphocytes, and monocytes were isolated from cryopreserved Ficoll- and CPT-isolated PBMC. RNA and DNA were extracted from total PBMC and immune cell subsets obtained after both Ficoll and CPT isolations. Next, gene expression was analyzed in immune cell subsets and total PBMC prepared by both methods. The general outline of the study is presented in Fig. 1.

### Isolation of PBMC by Ficoll-Paque density gradient centrifugation

PBMC were isolated from peripheral blood using Ficoll-Paque, as previously reported [34, 35]. Briefly, ACD-A-anticoagulated blood was centrifuged at 800 × *g* for 30 min and the top layer containing plasma was removed. The remaining blood was diluted with an equal volume of phosphate-buffered saline, pH 7.4 (PBS), containing 0.05 M ethylenediaminetetraacetic acid (EDTA; Invitrogen). 12.5 ml of diluted blood was layered over 25 ml of the Ficoll-Paque PLUS (GE Healthcare). Gradients were centrifuged at 400 × *g* for 30 min at room temperature in a swinging-bucket rotor without the brake applied. The PBMC interface was carefully removed by pipetting and washed with PBS-EDTA by centrifugation at 250 × *g* for 10 min. PBMC pellets were suspended in ammonium-chloride-potassium (ACK) lysing buffer (Invitrogen) and incubated for 10 min at room temperature with gentle mixing to lyse contaminating red blood cells (RBC). This was followed by a wash with PBS-EDTA. Cell number and viability were determined using a Countess Automated Cell Counter (Invitrogen). Non-viable cells were identified by staining with trypan blue and cell viability was calculated using the total cell count and the count of non-viable cells. PBMC were cryopreserved in liquid nitrogen in fetal calf serum (FCS; Invitrogen) containing 10 % dimethyl sulfoxide (DMSO; Thermo Fisher Scientific) and stored until required for downstream analyses.

### Isolation of PBMC using BD Vacutainer CPT

Immediately following blood collection with Vacutainer CPT, the tubes were inverted 10 times and centrifuged at 1,700 × *g* for 30 min at room temperature in a swinging-bucket rotor. After centrifugation, 3 ml of plasma was removed from the uppermost layer. The PBMC layer was gently suspended in the remaining plasma and transferred to 15-ml conical tubes and washed with PBS by centrifugation at 300 × *g* for 10 min. It is of note that an increase in RBC contamination compared to the Ficoll gradient method was visually observed, which was consistent with that reported by the manufacturer. Therefore, to remain consistent with the Ficoll protocol, PBMC pellets were re-suspended in ACK lysing buffer and incubated for 10 min at room temperature to lyse contaminating RBC and then washed with PBS by centrifugation. PBMC yield and viability were determined using a Countess Automated Cell Counter (Invitrogen) and cells were stored in liquid nitrogen as described above.

### Isolation of immune cell subsets

PBMC isolated by both the Ficoll and CPT methods predestined for cell subset isolations were removed from liquid nitrogen storage and thawed on wet ice. Cells were transferred to 15 ml conical tubes containing RMPI-1640 medium (Invitrogen) supplemented with 10 % FCS and 2 mM EDTA and recovered by centrifugation at 250 x *g* for 10 min at 4 °C. Then, PBMC were gently re-suspended in 10 ml RMPI-1640 supplemented with 10 % FCS and 10 mM EDTA, and incubated at 37 °C for 1 h to allow recovery of the cells prior to subset separation. PBMC number and viability were determined as indicated above.

Positive selection of immune cell subsets was performed by suspending recovered PBMC in 80 μl of autoMACS running buffer (MACS separation buffer; Miltenyi Biotec) per 1 x 10^7^ cells and by incubating with 22 μl antibody-labelled microbeads (Miltenyi Biotec) for 15 min at 4 °C. PBMC were washed with 10 ml of autoMACS running buffer by centrifugation at 250 × *g* for 10 min at 4 °C and thoroughly re-suspended in 2 ml of the same buffer to ensure a single-cell suspension. Separation of microbead-labelled cells was done using an autoMACS Pro Separator (Miltenyi Biotec). CD19+ (B cells), CD8+ (cytotoxic T cells), CD14+ (monocytes), and CD4+ (helper T cell) subsets were isolated from the same PBMC sample in sequential order using CD19, CD8, CD14, and CD4 microbeads, respectively. This isolation procedure was developed through an extensive series of preliminary experiments and found to be applicable for both freshly isolated and cryopreserved human PBMC (MacParland et al., manuscript in preparation). Separation of subsets from PBMC isolated from an individual donor on Ficoll gradient and by using CPT was performed simultaneously on two calibrated for compatibility autoMACS separators. Cell subset yield and viability were determined with a Countess Automated Cell Counter and compared between the two methods of PBMC isolation. Subset purity was determined using flow cytometric analysis, as described below. The isolated cell subsets were re-suspended in RLT Plus buffer (Qiagen) for subsequent RNA and DNA extractions.

### Flow cytometry

Purity of PBMC subsets was determined using flow cytometric analysis immediately following isolation of immune cell subsets. For each labelling, 2.0 × 10^5^ cells were washed with autoMACS running buffer as described above and re-suspended in 100 μl of autoMACS running buffer. Cells were incubated for 10 min on ice with human Fc receptor blocking reagent (Miltenyi Biotec), followed by 30 min incubation on ice with fluorochrome-labelled antibodies. CD19+, CD8+, CD14+, and CD4+ cell subsets were stained with allophycocyanin (APC)-conjugated anti-human CD19 (HIB19; eBioscience, San Diego, CA), Alexa Fluor 488-conjugated anti-human CD8 (OKT8; eBioscience), R-phycoerythrin (PE)-conjugated anti-human CD14 (61D3; eBioscience), and peridinin chlorophyll protein (PerCP)-Cy5.5-conjugated anti-human CD4 (RPA-T4; eBioscience), respectively. Incubations with matched immunoglobulin isotypes were done in parallel as controls using APC-conjugated mouse IgG1 (P3; eBioscience), Alexa Fluor 488-conjugated mouse IgG2a (eBM2a; eBioscience), PE-conjugated mouse IgG1 (P3; eBioscience), or PerCP-Cy5.5-conjugated mouse IgG1 (P3; eBioscience). After incubation with antibody, cells were washed twice with 2 ml of autoMACS buffer by centrifugation at 300 × *g* for 10 min at 4 °C, fixed in 2 % paraformaldehyde (Sigma) in PBS, washed again, and analyzed with a BD FACSCalibur bench top flow cytometer. The data were analysed using FlowJo 7.6 software (Flowjo, LLC).

### Extraction, quantitation, and qualitative analysis of RNA and DNA

RNA and DNA were simultaneously extracted from total PBMC or individual immune cell subsets, and purified using the AllPrep DNA/RNA Mini Kit (Qiagen) following the manufacturer’s instruction. RNA quantitation and quality were determined using an Agilent 2100 Bioanalyzer (Agilent and the RNA 6000 Nano Kit (Agilent), according to the manufacturer’s manuals. RNA quality was assessed using the RNA integrity number (RIN), a numerical score of the integrity of RNA, as reported by others [17, 36, 37]. The yield and purity of DNA were determined by spectrophotometric measurements of the ratio of UV absorbance at 260 and 280 nm by a Nanodrop 2000 (Thermo Fisher Scientific).

### Acquisition and analysis of gene expression data

The Affymetrix Human Genome U133 Plus 2.0 array GeneChips (Affymetrix, Inc.) were used for gene expression profiling and the evaluation was performed by Expression Analysis, Inc. The microarray contained more than 54,000 probe sets representing approximately 47,400 transcripts, allowing the analysis of over 38,500 genes. RNA samples were converted to amplified cDNA using the NuGEN Ovation RNA Amplification System version 2 (NuGEN) and subsequently labeled using the NuGEN Encore Biotin Module for hybridization onto the Affymetrix GeneChips. First strand cDNA was reverse transcribed from RNA (50 ng) using a DNA/RNA chimeric primer followed by second strand synthesis to generate double-stranded cDNA. Single primer isothermal amplification (SPIA) was then performed using a DNA/RNA chimeric primer, DNA polymerase, and RNase H. To generate labeled cDNA, 3.75 μg of purified SPIA-amplified cDNA was fragmented and labeled by enzymatic attachment of a biotin-labeled nucleotide to the 3-hydroxyl end. For hybridization, a hybridization cocktail was added to the labeled cDNA target using the Hybridization, Wash and Stain kit (Affymetrix, Inc.), applied to the microarrays, and incubated for 18 h at 45 °C. Following hybridization, the microarrays were washed and stained according to standard Affymetrix procedures before scanning on an Affymetrix GeneChip Scanner. The data were collected using Affymetrix Expression Console Software.

Microarray data were analyzed using Partek Genomics Suite software, version 6.6 (Partek Inc.) and Affymetrix Expression Console Software. The standard quality metrics of Partek and principle component analysis (PCA) were used for visualization of sample distribution to detect potential outliers [38]. To identify differentially expressed genes within a given cell type between the cells derived from PBMC isolated by the CPT and Ficoll methods, a two-group comparison with permutation analysis for differential expression, which provides an estimate of the false discovery rate (FDR) for each transcript comparison [39], was performed by an expert consultant (Expression Analysis, Inc.). Only transcripts that had a FDR less than 0.05 were defined as being significantly differentially expressed. When no significant differences were detected, transcripts with a FDR less than 0.1 were considered. The Affymetrix microarray data obtained during this study (30 samples) have been deposited in the NCBI’s Gene Expression Omnibus (GEO) [40] and are accessible through GEO Series accession number GSE67321 [http://www.ncbi.nlm.nih.gov/geo/query/acc.cgi?acc=GSE67321].

### Statistical analysis

Where indicated, the Student’s paired *t*-test (*P* < 0.05) accompanied by the Shapiro-Wilk test for normality (*P* < 0.05) were performed in order to compare PBMC yield and viability, immune cell subset yield and viability, and RNA and DNA yield and quality between the Ficoll and CPT PBMC isolation methods.

## Results

### Equivalent yield and viability of PBMC isolated using Ficoll or CPT protocols

**Fig. 2:**
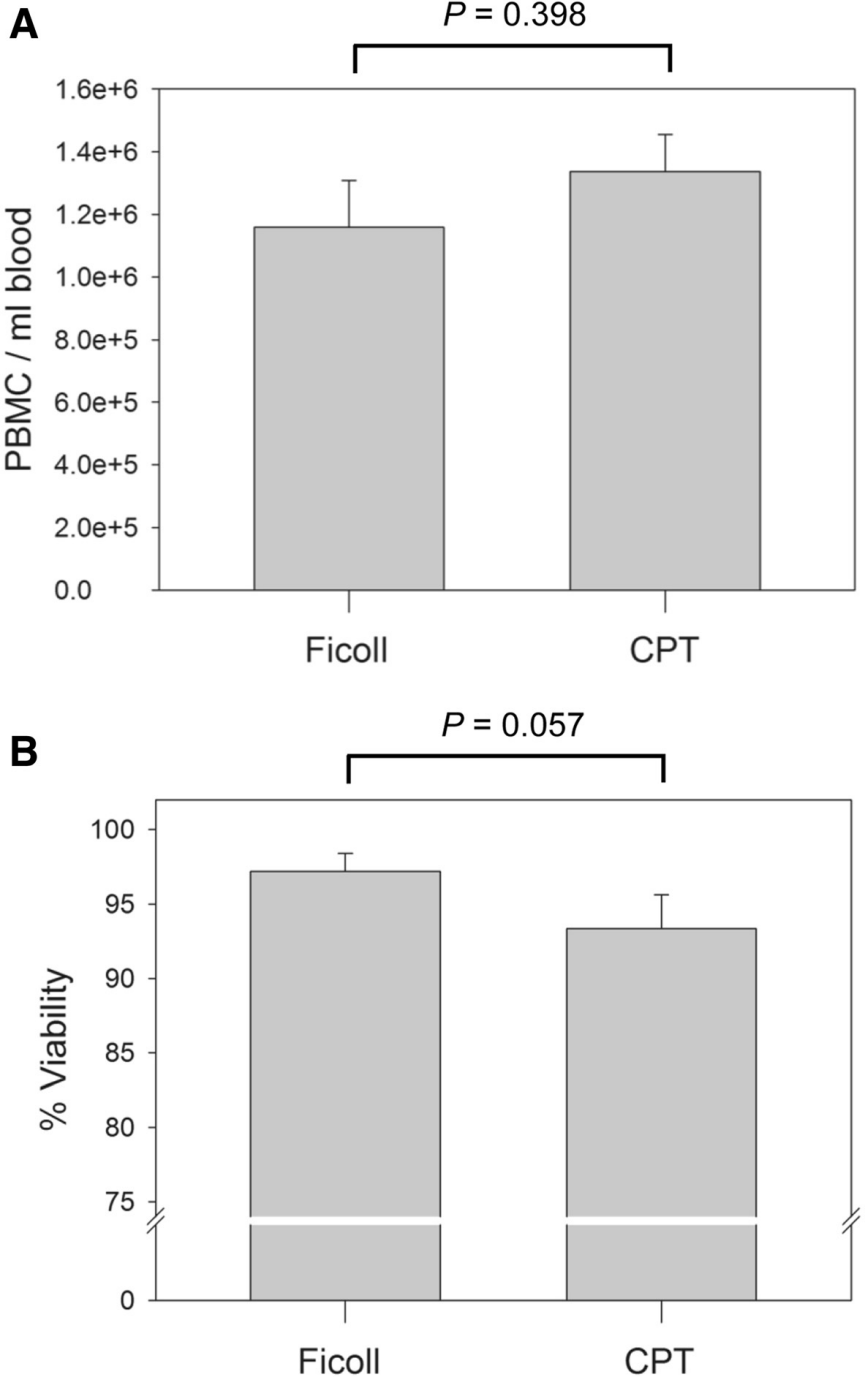
Yield and viability of PBMC isolated by the Ficoll and CPT protocols. **a** The mean numbers of PBMC per ml of blood obtained by Ficoll or CPT isolation procedure from 6 healthy donors. **b** The mean viability of PBMC freshly isolated from the same 6 healthy donors by either Ficoll gradient separation or CPT technique. No significant differences (*P* < 0.05) in PBMC yield or viability between the two isolation protocols were found. Error bars indicate standard error of the mean

The number and viability of PBMC from 6 healthy donors was assessed immediately following isolation by either the Ficoll or CPT technique and compared between the two. As described in the Methods, a RBC lysis step was included after purification of PBMC by both Ficoll and CPT protocols to minimize the impact of potentially contaminating RBC- and reticulocyte-derived RNA on downstream RNA and gene expression analyses. To correct for variation in the volume of blood used for PBMC isolation, a relative PBMC yield was calculated by dividing the total number of cells by the milliliters of blood from which the cells were isolated. The mean number of PBMC per ml of blood isolated by using the Ficoll method was 1.16 × 10^6^ cells/ml (SEM = 1.49 × 10^5^, range = 5.71 × 10^5^ – 1.67 × 10^6^) compared to 1.34 × 10^6^ cells/ml (SEM = 1.19 × 10^5^, range = 9.43 × 10^5^ – 1.71 × 10^6^) for the CPT technique (Fig. 2a). Thus, there was no significant difference in the number of PBMC isolated between the two methods (*P* = 0.398). The mean number of PBMC obtained per each CPT isolation (9.87 × 10^6^, SD = 3.71 × 10^6^, range = 4.95 × 10^6^ – 1.80 × 10^7^; *n* = 29) was comparable to that reported by the manufacturer (1.27 × 10^7^, SD = 4.64 × 10^6^, range = 7.02 × 10^6^ – 2.14 × 10^7^; *n* = 10). Both methods also resulted in isolation of similarly highly viable PBMC. The mean viability of Ficoll-isolated PBMC was 97.2 %, while that obtained by CPT method was 93.3 % (Fig. 2b) and this was not significantly different (*P* = 0.057).

### High purity and comparable viability of immune cell subsets derived from Ficoll- and CPT-isolated PBMC

**Fig. 3:**
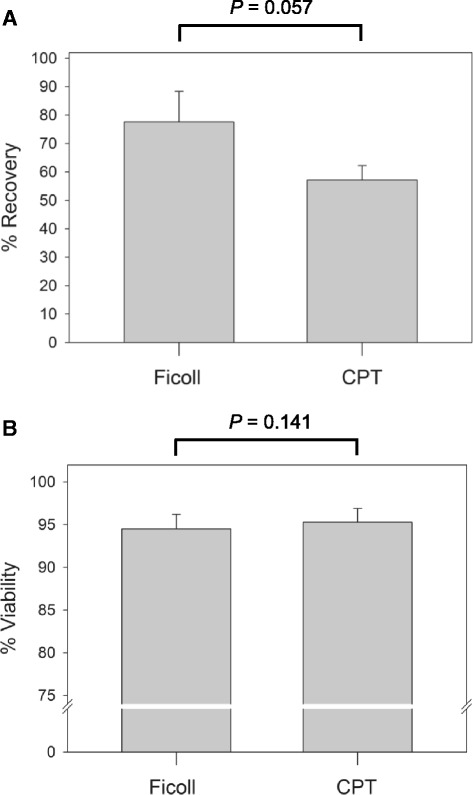
Post-cryopreservation recovery and viability of PBMC isolated by the Ficoll and CPT methods. **a** The mean percent recovery after cryopreservation of PBMC collected using the Ficoll and CPT protocols. Percent recovery was calculated by dividing the number of PBMC recovered after thawing by the number of cells that were cryopreserved. **b** The mean viability of recovered PBMC isolated by the Ficoll and CPT technique. No significant differences (*P* < 0.05) in percent recovery or viability between the two protocols were observed. Error bars represent standard error of the mean

Extensive evaluations performed prior to this study has resulted in the development of a standardized protocol allowing for the separation of highly pure B cells (CD19+), CD8+ T cells (CD8+), CD4+ T cells (CD4+) and monocytes (CD14+) from a single PBMC sample using an automated, magnetic bead-based, positive selection technique (MacParland et al., manuscript in preparation). Additional preliminary experiments showed that cryopreserved PBMC are a suitable source for positive selection of immune cells when compared to freshly isolated PBMC. The current study showed that the PBMC recovery from storage in liquid nitrogen was not significantly different (*P* = 0.057) when PBMC were isolated using either Ficoll or CPT (Fig. 3a). The mean percent recovery of PBMC obtained by Ficoll was 77.6 % (SEM = 10.7, range = 56.7–100.0) compared to 57.1 % (SEM = 5.1, range = 40.3–73.6) for CPT-isolated PBMC. Furthermore, the viability of recovered PBMC was excellent and did not significantly differ (*P* = 0.141) between Ficoll and CPT isolated cells, where the mean viabilities were 94.5 % (SEM = 1.7, range = 89.0–99.0 %) and 95.3 % (SEM = 1.6, range = 89.0–99.0 %) for Ficoll and CPT, respectively (Fig. 3b). These results were in agreement with previous reports that demonstrated similar post-cryopreservation recoveries and viability [18, 21, 22]. Taken together, the above results indicated that cell recovery from cryopreservation was not affected by the PBMC isolation method and supported our previous findings that cryopreserved PBMC are appropriate for isolation of immune cell subsets (MacParland et al., manuscript in preparation).

**Fig. 4:**
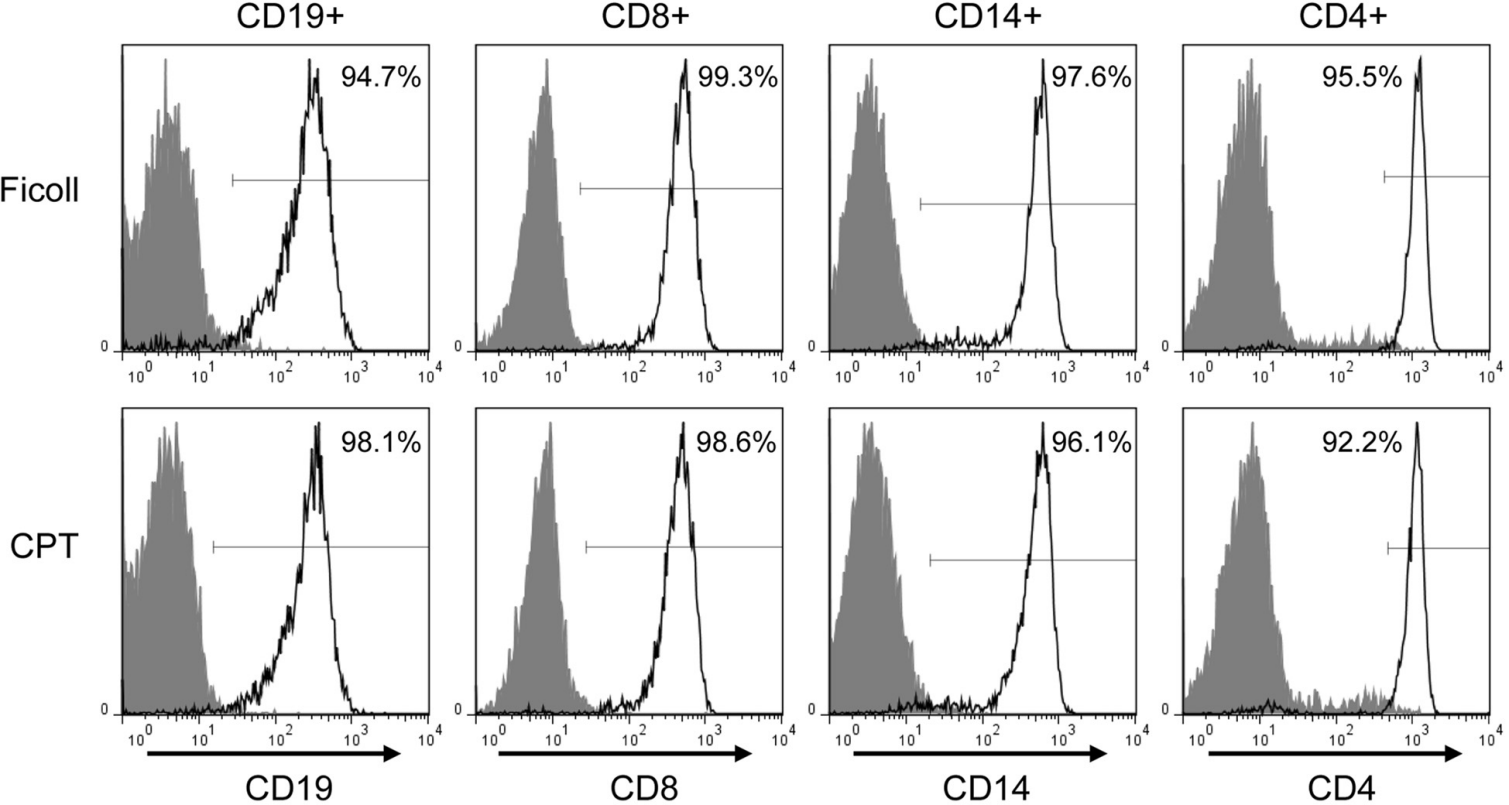
Purity of immune cell subsets obtained from PBMC isolated by either Ficoll or CPT procedures. CD19+, CD8+, CD14+, and CD4+ cell subsets were sequentially separated from PBMC isolated by Ficoll and CPT protocols from the same donor and their purity was determined by flow cytometry using antibodies against surface markers specific for individual immune cell types. Filled histograms were given by appropriate immunoglobulin isotype controls. Gates for determining positivity were established using isotype controls so that ~99.0 % of events were negative

Accordingly, CD19+, CD8+, CD14+, and CD4+ cells were sequentially separated from each PBMC sample examined and their quantity and viability compared. Purities of CD19+, CD8+, CD14+, and CD4+ cells were evaluated by flow cytometry and were found to be usually 95 % or greater for cells derived from PBMC isolated by either Ficoll or CPT, as shown for cells from one of the donors in Fig. 4.

**Fig. 5:**
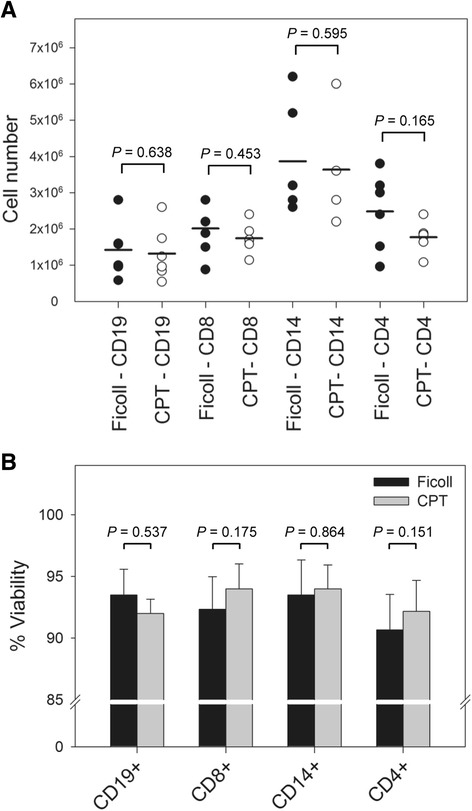
Yield and viability of immune cell subsets prepared from PBMC isolated by either Ficoll or CPT protocol. **a** The mean number of CD19+, CD8+, CD14+, and CD4+ cells positively selected from Ficoll- and CPT-isolated PBMC collected from 6 healthy donors. The horizontal line denotes the means and each symbol represents an individual cell type isolated from Ficoll-PBMC (filled symbols) or CPT-PBMC (empty symbols) from each of 6 donors. **b** The mean viability of the same cell subsets. No significant differences (*P* < 0.05) in yields and viability of the immune cell subsets obtained from PBMC isolated by Ficoll and CPT protocols were found. Error bars indicate standard error of the mean

Also, no significant differences were found when comparing the number or yield of CD19+, CD8+, CD14+, and CD4+ cells from PBMC separated by Ficoll or CPT protocol (Fig. 5a). The mean numbers of positively selected B lymphocytes were 1.42 × 10^6^ (SEM = 3.19 × 10^5^) and 1.32 × 10^6^ (SEM = 3.05 × 10^5^) for Ficoll- and CPT-derived PBMC, respectively (*P* = 0.638). The mean number of CD8+ T cells obtained from Ficoll-isolated PBMC was 2.01 × 10^6^ (SEM = 3.08 × 10^5^), while that from CPT- isolated PBMC was 1.74 × 10^6^ (SEM = 1.70 × 10^5^) (*P* = 0.453). Comparable (*P* = 0.595) numbers of monocytes were also acquired from PBMC prepared by both techniques, with a mean yield of 3.87 × 10^6^ (SEM = 6.01 × 10^5^) and 3.63 × 10^6^ (SEM = 5.28 × 10^5^) cells from Ficoll- and CPT-derived PBMC, respectively. Lastly, the mean yield of CD4+ T lymphocytes was 2.48 × 10^6^ (SEM = 4.39 × 10^5^) for Ficoll-PBMC compared to 1.77 × 10^6^ (SEM = 1.74 × 10^5^) for CPT-PBMC with no significant difference detected (*P* = 0.165). These results indicated that the employment of the CPT protocol for PBMC collection does not alter the reciprocal proportions between isolated immune cell subsets or the surface expression of immune cell-defining surface molecules in comparison to the isolation of PBMC by the Ficoll method.

Similar to yields of individual immune cell subsets, a comparison of viability between immune cells obtained from PBMC isolated by Ficoll and those by CPT method was assessed. These two PBMC isolation protocols did not result in any significant differences (Fig. 5b). Thus, the mean viabilities of CD19+, CD8+, CD14+, and CD4+ cells from Ficoll-PBMC were 93.5, 92.3, 93.5, and 90.7 % respectively, compared to 92.0, 94.0, 94.0, and 92.2 % for CPT-PBMC (*P* = 0.537, *P* = 0.175, *P* = 0.864, and *P* = 0.151, respectively). This clearly indicated that the CPT method of PBMC isolation does not adversely affect integrity of any of the PBMC subsets and that the use of either Ficoll or CPT for PBMC isolation in combination with the sequential immune cell isolation protocol provides viable cells for downstream evaluations, as the analysis of the gene expression profiles confirmed (see below).

### PBMC isolation method did not influence the quantity or quality of nucleic acids extracted

**Fig. 6:**
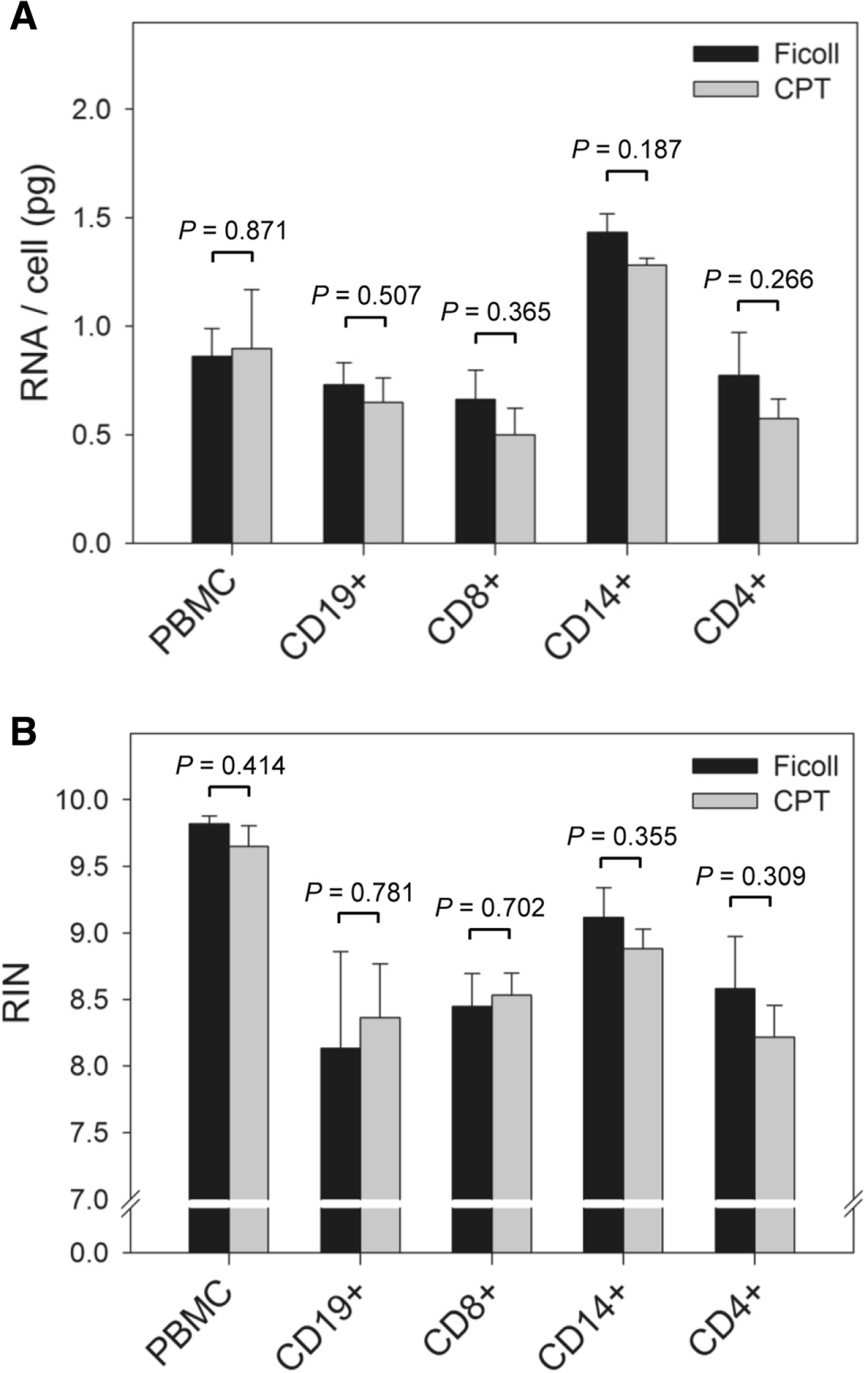
Yield and quality of RNA recovered from total PBMC and their individual immune cell subsets did not significantly differ between PBMC isolated by Ficoll and CPT methods. **a** The mean amount of RNA per cell and (**b**) the mean RIN of RNA preparations extracted from total PBMC and their individual immune cell subsets derived from PBMC prepared by Ficoll and CPT methods from 6 health donors. There were no significant differences (*P* < 0.05) in the amount of RNA obtained and in the quality of RNA, as measured by RIN, between the two methods of PBMC isolation*.* Error bars indicate standard error of the mean. RIN, RNA integrity number

**Fig. 7:**
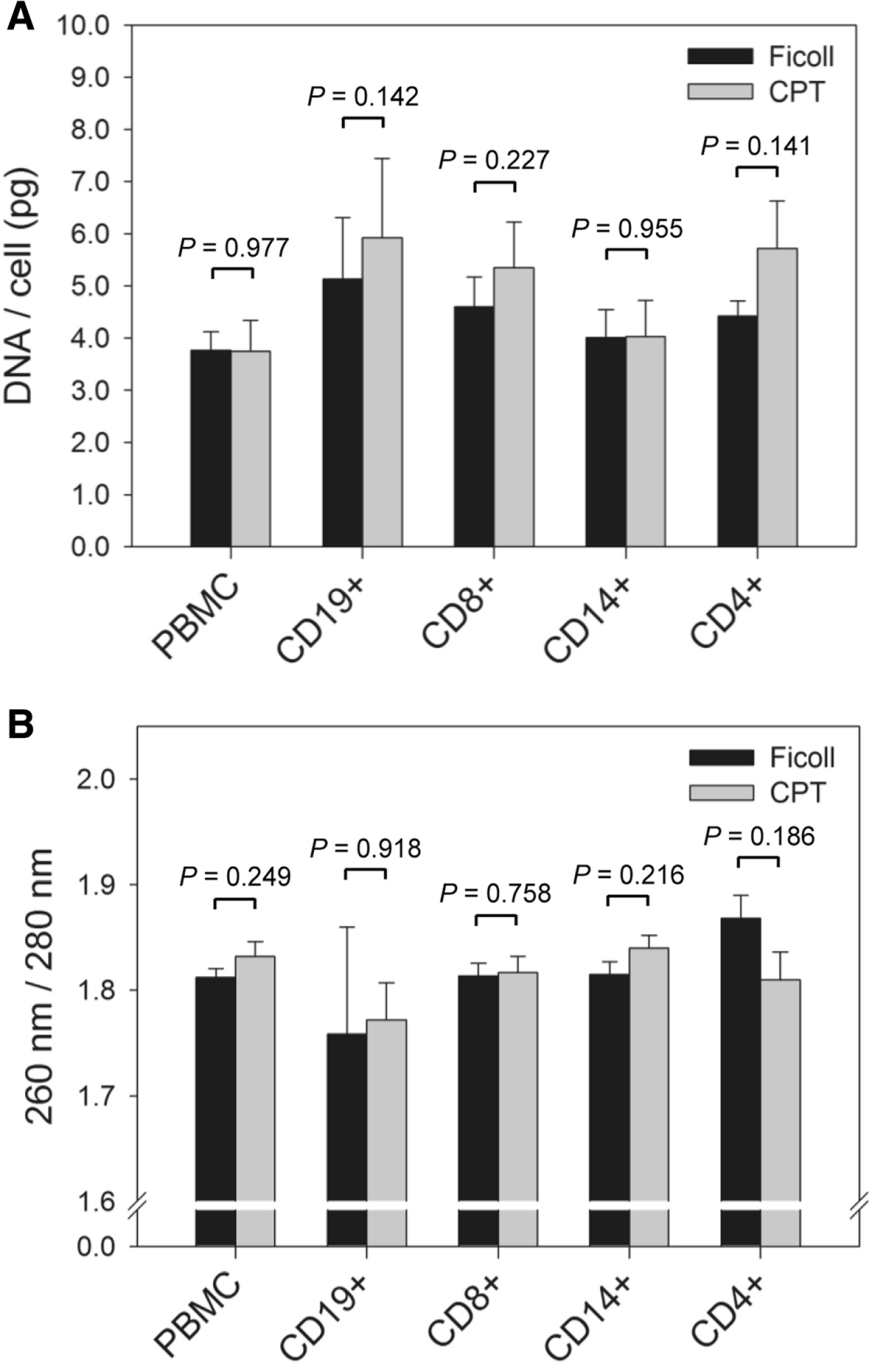
Similar quantities of DNA with comparable quality were obtained from PBMC and immune cell subsets following either Ficoll or CPT protocol of PBMC isolation. **a** The mean amount of DNA extracted per cell and (**b**) the mean of the 260/280 ratios, as measure of DNA quality, were compared between DNA preparations obtained from total PBMC and immune cell subsets prepared from PBMC isolated by Ficoll and CPT methods from 6 healthy donors. There were no significant differences (*P* < 0.05) in the amount of DNA recovered and in its quality between the two methods of PBMC isolation. Error bars indicate standard error of the mean

Since the yields of viable immune cells from PBMC isolated by Ficoll and CPT were similar, it was expected that this would also hold true when comparing the amounts of RNA and DNA extracted from these cells. As indicated, RNA and DNA were extracted from both total PBMC and individual immune cell subsets isolated from these PBMC. To compare yields, the amount of RNA and DNA was normalized to the number of cells used for extraction. The mean amount of RNA attained from total CPT-PBMC was 0.90 pg/cell, which was similar to 0.86 pg/cell for Ficoll-PBMC (*P* = 0.871) (Fig. 6a). The average yields of RNA from B cells derived from Ficoll- and CPT-PBMC were 0.73 pg/cell and 0.65 pg/cell, respectively (*P* = 0.507); CD8+ T lymphocytes, 0.66 pg/cell and 0.50 pg/cell, respectively (*P* = 0.365); monocytes, 1.43 pg/cell and 1.28 pg/cell, respectively (*P* = 0.187), and CD4+ T lymphocytes, 0.77 pg/cell and 0.57 pg/cell, respectively (*P* = 0.266) (Fig. 6a). Similar to RNA yields, there were no significant differences in the yields of DNA (Fig. 7a). Thus, the average amount of DNA extracted from Ficoll-PBMC, and CD19+, CD8+, CD14+ and CD4+ cells derived from these PBMC were 3.76, 5.13, 4.60, 4.01 and 4.42 pg/cell, respectively, compared to 3.74, 5.92, 5.35, 4.03, and 5.71 pg/cell for the same cells derived by CPT isolation protocol (*P* = 0.977, 0.142, 0.227, 0.955, and 0.141, respectively). Overall, the results indicated that the PBMC isolation method did not affect the number of separated immune cell subsets or the amount of nucleic acids extracted.

The integrity of RNA is of paramount importance for experiments that attempt to measure gene expression in biological samples when using Affymetrix microarrays. The RNA integrity is relatively less important when using RT-PCR and nanostring technology, although the downside with these technologies is the limitation to much smaller number of genes which expression can be analyzed simultaneously. In this study, the quality of extracted RNA from each cell sample was determined using the RNA RIN to compare whether collection of PBMC by CPT may augment RNA degradation in comparison to Ficoll. The average RIN of RNA preparations from total PBMC was nearly identical between Ficoll (9.82 ± 0.06) and CPT (9.65 ± 0.15) (*P* = 0.414), where a RIN equal to 10 indicates fully intact RNA (Fig. 6b). Likewise, the use of CPT for PBMC isolation did not affect the quality of RNA from B cells (Ficoll: 8.13 ± 0.73; CPT: 8.37 ± 0.40; *P* = 0.781); CD8+ T lymphocytes (Ficoll: 8.45 ± 0.25; CPT: 8.53 ± 0.16; *P* = 0.702); monocytes (Ficoll: 9.12 ± 0.22; CPT: 8.88 ± 0.14; *P* = 0.355), and CD4+ T lymphocytes (Ficoll: 8.58 ± 0.39: CPT: 8.22 ± 0.24; *P* = 0.309), when compared with the respective subsets derived from PBMC isolated on Ficoll (Fig. 6b).

The quality of DNA was assessed using the ratio of UV absorbance at 260 and 280 nm and compared between cell types prepared from Ficoll-PBMC and CPT-PBMC. The mean 260/280 ratios of DNA from total PBMC were equivalent between Ficoll (1.81) and CPT (1.83) preparations and did not significantly differ (*P* = 0.249) (Fig. 7b). The same was true for mean DNA 260/280 ratios for DNA isolated from CD19+ cells (Ficoll: 1.76; CPT: 1.77; *P* = 0.918), CD8+ cells (Ficoll: 1.81; CPT: 1.82; *P* = 0.758), CD14+ cells (Ficoll: 1.82; CPT: 1.84; *P* = 0.216), and CD4+ cells (Ficoll: 1.87; CPT: 1.81; *P* = 0.186) (Fig. 7b). Taken together, the above findings clearly demonstrated that the CPT protocol of PBMC isolation can substitute the Ficoll isolation of PBMC when immune cell subsets need to be purified without compromising quantity of the cells, and quantity and integrity of nucleic acids.

### PBMC isolation method did not affect the gene expression profile of total PBMC and immune cell subsets

**Fig. 8:**
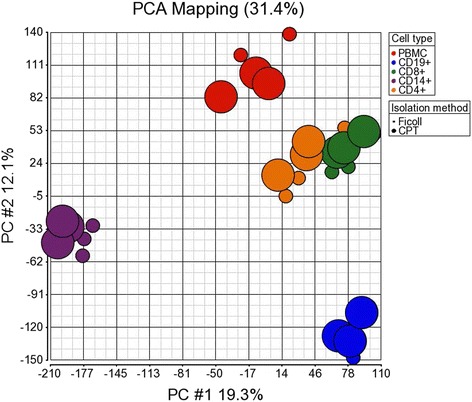
Principle component analysis of gene expression showing distinct clusters between individual immune cell subsets but not between the same subsets derived from PBMC isolated by CPT or Ficoll protocols. Principle component analysis (PCA) was performed based on expression of all genes from the microarray. Individual immune cell types are represented by different colors: CD19+ B cells, blue; CD8+ T cells, green; CD14+ monocytes, purple; CD4+ T cells, orange; and total PBMC, red. The method used for PBMC isolation is represented by different symbols of differing size: CPT-based procedure by large circles and Ficoll-based procedure by small circles. PC#1, principle component #1; PC#2, principle component #2

**Fig. 9:**
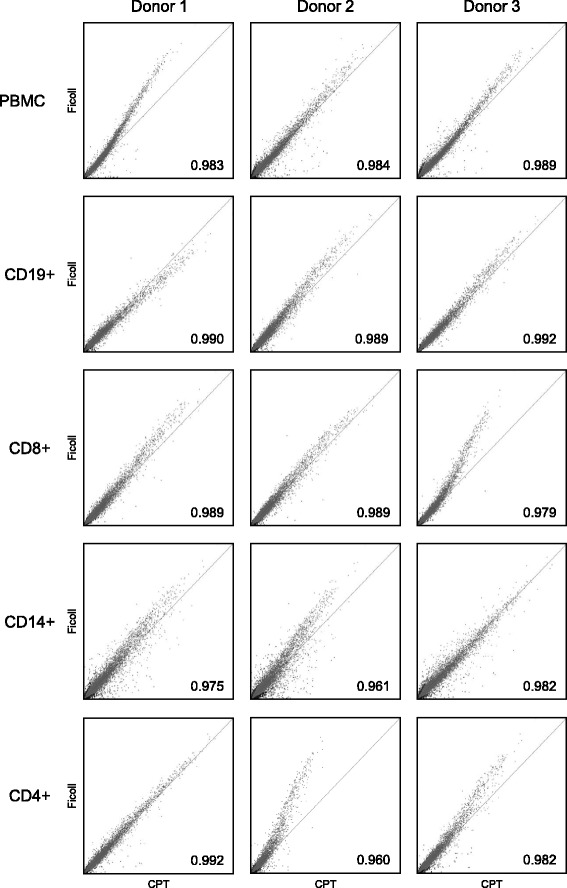
Signal scatter plots comparing the intensities of gene expressions on microarray chips within Ficoll and CPT-derived PBMC and their immune cell subsets for each healthy donor tested. The expression signals of Ficoll-derived cells were plotted against the expression signals of CPT-derived cells for each cell type and donor. A line of perfect correlation is indicated in all plots. Pearson’s correlation coefficients between each pair of samples are shown at the bottom right of each plot. The data showed that the pattern of gene expression was very similar between Ficoll and CPT-derived cell types, as indicated by the high correlation coefficients

Previous reports have demonstrated that experimental handling and manipulation, including the method of tissue collection and preparation, can adversely affect gene expression in primary cells and tissues, which can interfere with the biological interpretation of results [15–18]. To compare gene expression between cell subsets from PBMC prepared by Ficoll and CPT isolation procedures, gene expression profiles were generated on the Affymetrix Human Genome U133 Plus 2.0 Array. We first performed PCA using all genes to visualize the global distribution of gene expression. PCA is a mathematical technique used to determine the key sources of variation in a multidimensional dataset by reducing the dimensionality of the dataset by finding new variables, termed principle components (PC). Using these principle components, each sample can be characterized by a few values which can be plotted, thus allowing a visual assessment of differences and similarities between samples [38]. PCA has been widely used in the analysis and visualization of microarray data [41, 42]. In the PC#1 vs PC#2 plot shown in Fig. 8, it is clearly evident that the predominant variation in gene expression between samples was attributable to cell type. Samples of the same cell type formed distinct clusters away from other cell types in the PC#1 vs PC#2 plot, which accounted for 19.3 % and 12.1 % of the total variance, respectively (Fig. 8). Given their similar cellular onotology, both T cell subset samples, CD8+ and CD4+, were grouped together more closely than the other immune cell types, indicating a close relation in terms of gene expression. Interestingly, samples processed with either Ficoll or CPT, within a given cell type, were also found closely grouped. Further analyses of the signal scatter plots comparing the intensities of gene expression between total PBMC and individual immune cell subsets purified from these PBMC obtained by either Ficoll or CPT gave similar results as above. The pattern of gene expression also was very similar between Ficoll and CPT-derived PBMC and their immune cell subsets obtained from each donor, with Pearson’s correlation coefficients greater than 0.96 for all comparisons (Fig. 9). Taken together, these results showed that in regard to a given cell type there was little or no difference in gene expression between the two PBMC isolation methods.

To identify specific genes that were significantly differentially expressed between the Ficoll and CPT isolation protocols within a given immune cell type, a two-group comparison with permutation analysis for differential expression, which estimates the FDR for each comparison, was performed. Transcripts were considered to be differentially expressed when having a FDR less than 0.05. When no differentially expressed transcripts were detected at the FDR <0.05 level, the statistical stringency was reduced to a include transcripts having a FDR less than 0.1. This analysis resulted in no significant differences being detected in gene expression between Ficoll and CPT protocols for PBMC (Additional file 1: Table S1) and immune subsets (Additional file 2: Table S2; Additional file 3: Table S3; Additional file 4: Table S4; Additional file 5: Table S5). These results implied that there were no significant alterations in gene expression in PBMC and all immune cell types when CPT was substituted for Ficoll to isolate PBMC. We did not attempt to identify gene signatures that differentiated individual immune cell populations because this was not the focus of this study and this type of analysis has been done by others [43–47].

## Discussion

The evaluation of the transcriptome of PBMC in *ex vivo* or *in vitro* situations using high throughput technologies, such as gene expression microarrays, is frequently applied to advance our general understanding of biological processes and specific disease mechanisms in particular. The availability of the BD Vacutainer CPT method to isolate human PBMC offers a significant advantage over the Ficoll method in that it minimizes variations in technical processing, simplifies isolation procedure, and reduces the length of elapsed time before RNA isolation. Despite previous reports emphasizing the effect of different collection and preparation methods on gene expression in isolated cells of interest [15–18], the CPT method has not been comprehensively evaluated in comparison to other blood cell collection techniques. In the current study, a direct comparison between the CPT and Ficoll method of PBMC isolation was done to determine if one method may have a significant advantage over the other in regard to the immune cell yield, viability, recovery of their individual cell types, quality of extracted nucleic acids, and gene expression in both total PBMC and positively-selected immune cell subsets.

Previous reports which have evaluated the yield of PBMC using the CPT method in comparison to the Ficoll technique have been inconsistent in their findings [21–23]. As in the present study, Ruitenberg et al. observed statistically equivalent numbers of cells isolated using Ficoll (2.98 × 10^6^ cells/ml) and CPT (2.68 × 10^6^ cells/ml) [21]. Another study also found similar yields by these two methods at one clinical site (Ficoll, 1.27 × 10^6^ cells/ml; CPT, 1.36 × 10^6^ cells/ml), but at a second site reported similar, although statistically significantly fewer PBMC numbers after CPT isolation (1.34 × 10^6^ cells/ml) compared to Ficoll (1.58 × 10^6^ cells/ml), suggesting that technical ability of personnel may have an impact on sample collection and/or cell recovery [22]. In contrast, Schlenke et al. reported that the CPT method resulted in significantly higher yields of isolated PBMC compared to the Ficoll isolation protocol [23]. These differences are likely due to some variations in technical expertise, protocols and reagents used in each study. For example, our CPT procedure incorporated a red blood cell lysing step followed by additional washes which may have contributed to the slightly lower yields compared to others [21, 48]. There was a trend, although not statistically significant, toward higher cell viability using Ficoll-isolated PBMC. Despite this minor difference, our findings supported the above studies in that the variability of cells isolated using either the CPT or Ficoll method was consistently greater than 90 % [21, 22].

While cell viability and yield are important characteristics, perhaps even more important is determination whether CPT- and Ficoll-isolated PBMC display comparable phenotypic profiles and biological functions. In this regard, it has been documented that T lymphocytes derived from CPT-PBMC exhibit similar responses to antigenic stimulation as PBMC isolated on Ficoll in interferon-gamma release immunoassays [21, 22, 48]. Furthermore, CPT-PBMC were equally suitable for serological typing of human leucocyte antigen (HLA) class I and detection of HLA antibodies using the complement-mediated microcytotoxicity technique when compared to Ficoll-PBMC [23]. They were also found suitable for the quantification of HIV RNA, HIV p24 antigen, and HIV anti-viral drug levels [49–51]. Taken together, the studies utilizing total PBMC isolated via CPT protocol showed that their phenotype and the spectrum of biological activities investigated so far were highly compatible with those of PBMC prepared using the Ficoll method.

The focus of this study was to compare the phenotype of Ficoll- and CPT-isolated PBMC in terms of their major immune subset makeup and corresponding gene expression. Earlier studies have demonstrated that variations in conventional PBMC isolation techniques can lead to the selective loss of certain cell subtypes [52–54]. For example, Schlenke et al. observed no difference in granulocyte contamination between CPT and Ficoll isolated PBMC if samples were immediately processed, but found that after a delay in processing, CPT were better at preventing granulocyte contamination, at the expense of decreasing T a B lymphocyte viability in comparison to Ficoll-PBMC [23]. Given that comparable numbers of PBMC were isolated by the two methods in our study, it was not surprising that no significant differences were observed in the number of highly pure, positively-selected B cells (CD19+), T cells (CD4+ and CD8+), and monocytes (CD14+) between CPT- and Ficoll-isolated PBMC (Fig. 4; Fig. 5a). Furthermore, the viability of immune subset cells derived from PBMC isolated by CPT and Ficoll protocols was consistently greater than 90 % (Fig. 5a). Although the absolute numbers of immune subsets were not determined in the total PBMC populations, the results presented here indicate that the proportion of the major immune subsets is not influenced by the PBMC collection method examined.

Comparable yields of RNA were purified from individual immune cell subsets from Ficoll- and CPT-isolated PBMC (Fig. 6a), which was expected given the equivalent yield of these cells. The mean amount of RNA extracted from total CPT-PBMC and Ficoll-PBMC was 0.90 and 0.86 pg/cell, respectively, almost identical to an earlier study which reported an RNA yield of 0.90 pg/cell for PBMC [17]. RNA integrity was maintained throughout the separation of immune subsets from both CPT- and Ficoll-PBMC, as evidenced by the high RIN values (Fig. 6b), which also reflected the high viability of separated subsets (Fig. 5b). In general, the quality of RNA was marginally reduced in the isolated immune cell subsets compared to total PBMC (RIN > 9.5 vs. > 8.0, respectively), likely due to the effect of the cryopreservation and thawing performed before the subset separation protocol. However, RNA integrity was still well above the acceptable cut-off RIN of 5.0 for the reliable gene expression quantitation, as suggested by earlier studies [17, 55].

Several studies have made use of CPT to isolate PBMC for the analysis of gene expression by qRT-PCR and microarray techniques [33, 56–61], but few have directly compared gene expression of CPT-PBMC with alternative methods of PBMC isolation. This is of particular importance given that the method of PBMC purification can adversely affect gene expression, especially when compounded with additional confounding variables [16, 33]. Whole blood RNA stabilization products such as PAXgene (Qiagen) and Tempus (Thermo Fisher Scientific) minimize *ex vivo* manipulation compared to leukocyte isolation techniques. However, whole blood contains large populations of granulocytes, erythrocytes, and platelets that contribute to gene expression, which can add a considerable amount of noise when the cells of interest are of lymphoid origin [62] and can significantly alter the gene expression profile when compared with isolated PBMC [45]. Ultimately, the use of whole blood RNA stabilization techniques or PBMC isolation methods will be determined by the nature of the scientific questions posed by a particular study, and the decision to employ CPT will follow this initial judgement. Although limited in the sample size, the current study did not detect any significant differentially expressed genes between PBMC purified by the Ficoll and CPT method using the Affymetrix HG-U133 Plus 2.0 microarray. In comparison, Baechler et al. used qRT-PCR to compare expression of commonly transcribed lymphoid cell genes in PBMC isolated using CPT or Ficoll and observed comparable levels of expression when samples were immediately processed [33]. Another study also found very few differences in the gene expression profiles of CPT- and Ficoll-PBMC measured using Affymetrix HG-U133A microarrays [61]. Together with these earlier findings, the results presented in this study indicate that gene expression in total PBMC remain unaltered irrespective of the isolation method employed.

One of the greatest sources of variation in PBMC gene expression among subjects is differences in the proportions of mononuclear cell subpopulations [45, 58]. This variation, which is known to differ based on age, sex, disease status, as well as other factors [26–28], adds an additional layer of complexity to the analysis of microarrays when they are used to compare the gene expression profile of PBMC between different biological situations, and subtle but specific changes in a minor immune subset may be overlooked. In addition, if differentially expressed genes are detected in a mixed cell population, such as PBMC, it is difficult to identify which cell type is responsible for the change detected and this confounds interpretation of the biological significance of the finding. Strategies have been developed to ascertain the extent to which changes in gene expression in a particular immune cell type can be detected from a total PBMC population [46, 63–69]. These gene deconvolution methods are able to discriminate gene expression of particular cell types within complex tissues and cell mixtures. They are relatively accurate when working with well-defined cell mixtures such as blood. However, they are much less reliable for mixtures with unknown composition and for discriminating between closely related cell phenotypes. Furthermore, deconvolution methods are limited in that they rely on the specificity of reference gene profiles, which may differ in cells and/or tissues in different disease states. Thus, the isolation of specific cell subsets prior to analysis of gene expression offers a distinct advantage by providing insight into immune cell subset-specific changes. For this reason, we separated B lymphocytes, CD8+ and CD4+ T lymphocytes, and monocytes from both Ficoll- and CPT-isolated PBMC to precisely determine whether the PBMC isolation method had a preferential effect on the immune cell subsets isolation and their gene expression signature which may have been masked by the analysis of total PBMC. The results revealed that there were no significant differences in gene expression in any of the immune cell subsets separated from Ficoll- and CPT-isolated PBMC. Although the current analysis of gene expression did not include RNA-seq data, it provides a basis of comparison for future evaluations that may utilize this method. Overall, our findings document that either method evaluated can be substituted for the collection of PBMC without adversely affecting subsequent immune cell subset separation and the gene expression by the resulting subsets.

## Conclusions

The present study demonstrates that the Ficoll isolation of PBMC can be substituted by the BD Vacutainer CPT protocol without yielding phenotypic changes in PBMC-derived immune cell subsets and without compromising the quality of the subsequent gene expression studies. Given that the CPT protocol is less elaborate, minimizes the cells’ handling and processing time, the current findings indicate that this method offers a significant operating advantage, especially in large-scale clinical studies aiming at dissecting gene expressions in total PBMC and their immune cell subset populations.

## Availability of supporting data

The microarray data files supporting the results presented in this article are available in the NCBI’s Gene Expression Omnibus and are accessible through GEO Series accession number GSE67321 [http://www.ncbi.nlm.nih.gov/geo/query/acc.cgi?acc=GSE67321].

The data sets supporting the results of this article are included within the article and its additional files.

## Additional files

Additional file 1: Table S1. Comparison of gene expression of total PBMC isolated using CPT and Ficoll. A two-group comparison with permutation analysis for differential expression was performed, as described in Methods, on the microarray data to identify differentially expressed genes between PBMC isolated by the CPT and Ficoll methods. Probsets are ranked according to the FDR value. File format: CSV (CSV 26347 kb) Additional file 2: Table S2. Comparison of gene expression of CD19+ B cells separated from PBMC isolated using CPT and Ficoll. A two-group comparison with permutation analysis for differential expression was performed, as described in Methods, on the microarray data to identify differentially expressed genes between CD19+ B cells separated from CPT- and Ficoll-isolated PBMC. Probsets are ranked according to the FDR value. File format: CSV (CSV 26341 kb) Additional file 3: Table S3. Comparison of gene expression of CD8+ T cells separated from PBMC isolated using CPT and Ficoll. A two-group comparison with permutation analysis for differential expression was performed, as described in Methods, on microarray data to identify differentially expressed genes between CD8+ T cells separated from CPT- and Ficoll-isolated PBMC. Probsets are ranked according to the FDR value. File format: CSV (CSV 26350 kb) Additional file 4: Table S4. Comparison of gene expression of CD14+ monocytes separated from PBMC isolated using CPT and Ficoll. A two-group comparison with permutation analysis for differential expression was performed, as described in Methods, on microarray data to identify differentially expressed genes between CD14+ monocytes separated from CPT- and Ficoll-isolated PBMC. Probsets are ranked according to the FDR value. (CSV 26353 kb) Additional file 5: Table S5. Comparison of gene expression of CD4+ T cells separated from PBMC isolated using CPT and Ficoll. A two-group comparison with permutation analysis for differential expression was performed, as described in Methods, on microarray data to identify differentially expressed genes between CD4+ T cells separated from CPT- and Ficoll-isolated PBMC. Probsets are ranked according to the FDR value. (CSV 26355 kb)

## Abbreviations

BD: Becton Dickinson
CPT: Cell preparation tube
ACD-A: Acid-citrate-dextrose anticoagulant, solution A
min: minutes
EDTA: Ethylenediaminetetraacetic acid
PBS: Phosphate-buffered saline, pH 7.4
RBC: Red blood cells
FCS: Fetal calf serum
DMSO: Dimethyl sulfoxide
ACK: Ammonium-chloride-potassium
APC: Allophycocyanin
PE: R-phycoerythrin
PerCP: Peridinin chlorophyll protein
SPIA: Single primer isothermal amplification
cDNA: Complementary DNA
NCBI: National Center for Biotechnology Information
GEO: Gene Expression Omnibus
PC: Principle component
SEM: Standard error of the mean
RIN: RNA integrity number
FDR: False discovery rate
HLA: Human leucocyte antigen
qRT-PCR: Quantitative reverse transcription polymerase chain reaction
LN_2_: Liquid nitrogen
